# Stress native T1 and native T2 mapping compared to myocardial perfusion reserve in long-term follow-up of severe Covid-19

**DOI:** 10.1038/s41598-023-30989-y

**Published:** 2023-03-13

**Authors:** Jannike Nickander, Rebecka Steffen Johansson, Klara Lodin, Anton Wahrby, Daniel Loewenstein, Judith Bruchfeld, Michael Runold, Hui Xue, Peter Kellman, Henrik Engblom

**Affiliations:** 1 Department of Clinical Physiology, Karolinska Institutet, Karolinska University Hospital, Stockholm, Sweden; 2 Division of Infectious Diseases, Department of Medicine Solna, Karolinska Institutet, Solna, Sweden; 3 Department of Infectious Diseases, Karolinska University Hospital, Stockholm, Sweden; 4 Department of Respiratory Medicine and Allergy, Karolinska University Hospital, Stockholm, Sweden; 5 National Heart, Lung, and Blood Institute, National Institutes of Health, Bethesda, MD USA

**Keywords:** Cardiology, Cardiovascular diseases, Infectious diseases

## Abstract

Severe Covid-19 may cause a cascade of cardiovascular complications beyond viral pneumonia. The severe inflammation may affect the microcirculation which can be assessed by cardiovascular magnetic resonance (CMR) imaging using quantitative perfusion mapping and calculation of myocardial perfusion reserve (MPR). Furthermore, native T1 and T2 mapping have previously been shown to identify changes in myocardial perfusion by the change in native T1 and T2 during adenosine stress. However, the relationship between native T1, native T2, ΔT1 and ΔT2 with myocardial perfusion and MPR during long-term follow-up in severe Covid-19 is currently unknown. Therefore, patients with severe Covid-19 (n = 37, median age 57 years, 24% females) underwent 1.5 T CMR median 292 days following discharge. Quantitative myocardial perfusion (ml/min/g), and native T1 and T2 maps were acquired during adenosine stress, and rest, respectively. Both native T1 (R^2^ = 0.35, p < 0.001) and native T2 (R^2^ = 0.28, p < 0.001) correlated with myocardial perfusion. However, there was no correlation with ΔT1 or ΔT2 with MPR, respectively (p > 0.05 for both). Native T1 and native T2 correlate with myocardial perfusion during adenosine stress, reflecting the coronary circulation in patients during long-term follow-up of severe Covid-19. Neither ΔT1 nor ΔT2 can be used to assess MPR in patients with severe Covid-19.

## Introduction

The novel betacoronavirus SARS Coronavirus 2 has resulted in a global pandemic of coronavirus disease 2019 (Covid-19)^1^, and was primarily associated with respiratory disease and systematic inflammation as the main cause of morbidity and mortality. However, there is increasing evidence linking Covid-19 with cardiovascular disease (CVD)^2^. Covid-19 infection may induce endothelial dysfunction, microvascular inflammation and thrombosis via angiotensin converting enzyme 2 and secondary autoimmune responses, causing coronary microvascular dysfunction (CMD), which might serve as a mechanism for long-term CVD post-Covid-19^3^. Myocardial perfusion during rest and pharmacological stress can be used to calculate the myocardial perfusion reserve (MPR) to assess the coronary circulation^4^, which can be performed with quantitative cardiovascular magnetic resonance (CMR) myocardial perfusion maps with an excellent agreement with positron emission tomography (PET)^5,6^.

Quantitative parametric pixelbased mapping by CMR has been developed to image both the longitudinal relaxation time constant (T1) and transverse relaxation time constant (T2)^7^. Both native T1 and T2 maps have been shown to identify acute myocardial inflammation causing edema and chronic pathologies with expanded interstitium where free water can distribute, and can be used for a range of myocardial pathologies^8^. Both native myocardial T1 and T2 depend on myocardial blood T1 and T2, which constitute a basis for T1 and T2 mapping to capture change in myocardial perfusion during stress without contrast agents^9,10^. Theoretically, the relative change in native T1 and T2, also called T1- and T2-reactivity (ΔT1 and ΔT2) (%), should portray the same physiology as MPR^11^. However, the relationship between native T1, native T2, ΔT1 and ΔT2 with myocardial perfusion and MPR during long-term follow-up in severe Covid-19 is currently unknown. Therefore, the aim of this study was to elucidate the relationships of the parameters native T1, native T2, ΔT1 and ΔT2 with quantitative myocardial perfusion and MPR during long-term follow-up in severe Covid-19 using CMR.

## Methods

### Study population

Patients hospitalized at Karolinska University Hospital, Stockholm, due to severe Covid-19 (n = 40, age median 57 interquartile range [IQR] [50–65], 23% females), were included from the project ”Follow-up of patients with severe Covid-19 pneumonia” (UppCov), aiming to characterize the long-term consequences of severe Covid-19 pneumonia in a comprehensible fashion. The patients underwent CMR at 1.5 T during long-term follow-up, between November 2020 and September 2021. Patients were eligible to be included in UppCov if discharged from the hospital at the intensive care unit and/or hospital wards for severe Covid-19, defined as respiratory failure with a higher demand of ventilatory support and oxygen (at least 5 l/min of oxygen flow rate).

Exclusion criteria for this CMR substudy included risk factors such as diabetes mellitus, myocardial infarction, aortic stenosis (AS), uncontrolled severe hypertension, atrial fibrillation and previous stroke. Patients that had undergone percutaneous coronary intervention and/or coronary artery bypass grafting or valvular surgery, or had contraindications for adenosine including chronic obstructive pulmonary disease and asthma or other standard safety contraindications such as renal failure or pacemaker were also excluded. Ethical approval was granted for all study procedures and all subjects provided written informed consent.

### Image acquisition

**Figure 1:**
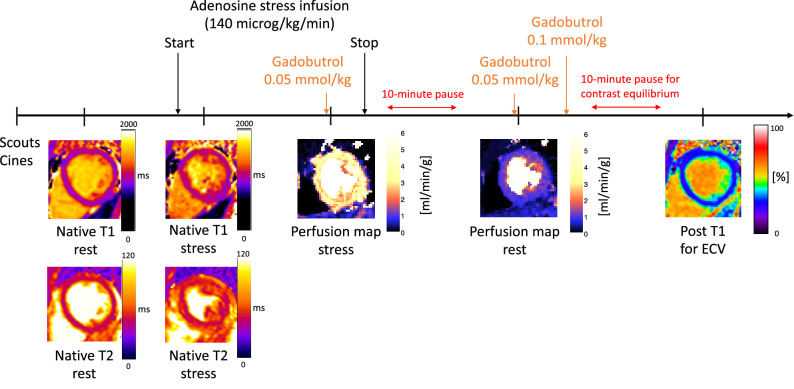
Image acquisition protocol. Scouts and cines were acquired first, followed by native T1 and native T2 mapping. The adenosine infusion was started, and after 3 min one native midventricular T1 map and T2 map was acquired prior to quantitative first pass perfusion imaging, following a bolus of contrast agent. The adenosine infusion was then terminated and after 10 min rest perfusion maps were acquired. Post contrast T1 maps for extracellular volumes maps were acquired after an additional 10 min. Modified from^8^.

CMR was performed at 1.5 T (MAGNETOM Aera, Siemens Healthcare, Erlangen, Germany). The image acquisition protocol is summarized in Fig. 1. Full coverage retrospective ECG-gated balanced steady state free precession (SSFP) cine imaging was acquired in short-axis and three long-axis slices. Typical imaging parameters were flip angle (FA) 68 degrees, pixel size 1.4 × 1.9 mm^2^, slice thickness 8.0 mm, echo time (TE)/repetition time (TR) 1.19 ms/37.05 ms, matrix size = 256 × 144 and field of view (FOV) 360 × 270 mm^2^.

Three short-axis slices (basal, midventricular, apical) were acquired using first-pass perfusion imaging rendering myocardial quantitative perfusion (ml/min/g) maps^5^, both during adenosine stress (Adenosin, Life Medical AB, Stockholm, Sweden, 140 microg/kg/min infusion) and at rest, following administration of an intravenous bolus of contrast agent (0.05 mmol/kg, gadobutrol, Gadovist, Bayer AB, Berlin, Germany). Perfusion maps were generated using the Gadgetron inline perfusion mapping software, freely available as an executeable^12^, computed based on a bistributed tissue exchange model^13^ that estimates arterial delay, perfusion, myocardial blood volme, permeability surface area and interstitial volume in resonable agreement with T1-based estimates of ECV^12^. Adenosine and contrast were administered in separate cannulas. Typical imaging parameters were: flip angle 50°, slice thickness 8.0 mm, TE/TR 1.04 ms/2.5 ms, bandwidth 1085 Hz/pixel, FOV 360 × 270 mm^2^ and saturation delay/trigger delay (TD) 105/40 ms.

Five short-axis native T1 maps were obtained during rest using an ECG-gated modified look-locker inversion (MOLLI, 5 s(3 s)3 s) recovery prototype sequence. One midventricular short axis T1-map was acquired during adenosine stress. Typical imaging parameters included single shot SSFP in end-diastole, flip angle 35 degrees, pixel size 1.4 × 1.9 mm^2^, slice thickness 8.0 mm, imaging duration 167 ms, TE/TR 1.12 ms/2.7 ms, matrix size = 256 × 144 and FOV 360 × 270 mm^2^. Five ECV-maps at rest were generated from native T1-maps and post-contrast T1-maps and calibrated by the hematocrit^14,15^.

Five short-axis native T2 maps were acquired before adenosine stress, and one midventricular short axis T2-map during adenosine stress. T2-mapping was performed using a T2-prepared sequence. Typical imaging parameters included TE/TR 1.06 ms/2.49 ms, FA 70 degrees, pixel size 1.8 × 1.8 mm^2^, slice thickness 8.0 mm, acquisition window 700 ms, TD 483 ms and matrix size = 144 × 256.

### Image analysis

**Figure 2:**
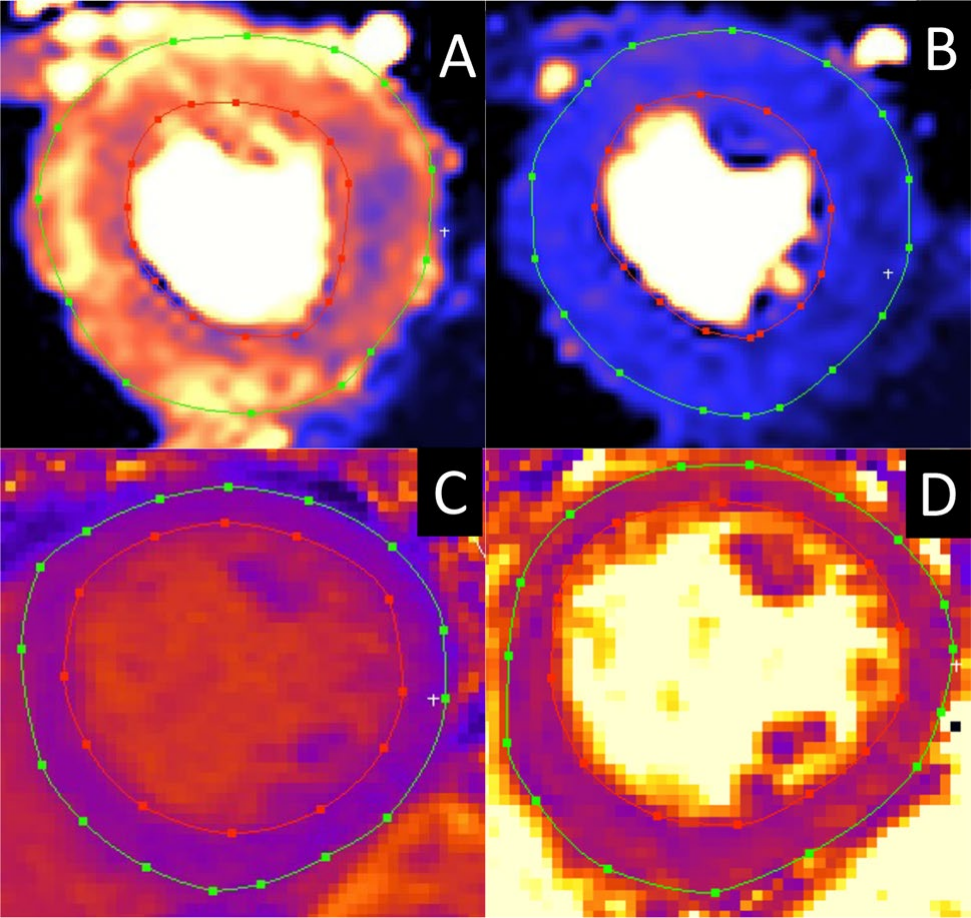
Examples of segmentations of endo- and epicardial borders. The image shows segmentations of the respetives maps: (**A**) stresss perfusion map, (**B**) rest perfusion map, (**C**) rest native T1 map and (**D**) rest native T2 map.

Cine images, quantitative perfusion maps, ECV-maps, T1- and T2-maps were analyzed with the software Segment^16^ (version 2.7 Medviso AB, Lund, Sweden) by carefully delineating the endo- and epicardial borders of the LV in the short-axis images, Fig. 2. To further avoid contamination from blood pool and adjacent tissues, respectively, a 10% erosion margin within Segment was set for both endo- and epicardial borders for the export of the respective mapping values. Global native T1 rest, native T2 rest, myocardial perfusion rest, myocardial perfusion stress and ECV values were acquired by averaging all segments of a 16-segment model of the LV. Global native T1 stress, and native T2 stress were averaged from a 6-sector bullseye plot. Intra- and inter-observer variability was assessed on the parameters myocardial rest perfusion, rest native T1 and rest native T2. For intra-observer variability one observer re-analyzed 10 subjects, while inter-observer variability analysis was performed on all 37 subjects by two independent observers.

### Statistical analysis

Statistical analysis was performed using Microsoft Excel version 16 (Microsoft, Redmond, Washington, USA) and IBM SPSS Statistics (IBM SPSS Statistics 27, IBM, New York, USA). All data was assessed for normality using the Kolmogorov–Smirnov test. Continuous data was expressed as median and IQR and categorical data was presented as numbers and percentages. Quantification of myocardial perfusion, ECV, native T1 and T2 was performed in each slice, and global values per subject were acquired by averaging the values from all short-axis slices in each subject. MPR was calculated as the ratio of stress to rest myocardial perfusion (ml/min/g). ∆T1 (%), and ∆T2 (%) were calculated as (stress-rest)/rest × 100 (%) for native T1 and T2, respectively. The individual relationships at rest and stress between parameters of myocardial perfusion, MPR and parameters of native T1, native T2, ∆T1 and ∆T2, were assessed by linear regression. By combining rest and stress values in the same data set to maintain aggregated data for T1, T2 and myocardial perfusion, the relations between these pooled parameters were investigated. Inter- and intra-observer agreement was calculated for myocardial rest perfusion, MPR, rest native T1, rest native T2, ∆T1 and ∆T2, and presented as intra-class correlation coefficient (ICC). The significance level in all statistical analyses was defined as p < 0.05.

### Ethics, consent and permission

All study procedures were carried out in accordance with relevant guidelines and regulations as per the Declaration of Helsinki and Good Clinical Practice for involving human participants. The study was approved by the Swedish Ethical Review Authority, Dnr 2020-04329 and all patients provided written informed consent.

## Results

### Study population

In total, 3 patients did not undergo adenosine stress CMR and were therefore excluded from the analysis. The remaining patients (n = 37) underwent adenosine-stress CMR scan 292 [207–367] days after discharge. All images were assessed with regards to image quality, and no patients were excluded due to poor image quality. Due to operator dependency, 1 patient did not obtain T2 maps and 1 patient did not obtain T2 maps at stress and were consequently excluded from analysis of native T2. Minimal (< 1 segment) late gadolinium enhancement (LGE) was found in 4 patients, and these patients were included in the analysis due to the limited extent of scarring. Baseline characteristics, including previous medical history, are presented in Table 1. CMR-findings are presented in Table 2, and stress findings in Table 3.

**Table 1:** Clinical characteristics of patients.

Clinical characteristics	n = 37
Female sex, n (%)	9 (24)
Age, years	57 [51–65]
Body height, cm	175 [170–180]
Weight, kg	86 [80–100]
BSA, m^2^	2.1 [2.0–2.3]
Creatinine, mmol/l	85 [69–101]
EVF, %	41 [40–47]
Hs-TnT, ng/l	51 [23–183]
PAP, mmHg^a^	50 [40–55]
Diabetes mellitus, n (%)	1 (2.7)
Atrial fibrillation, n (%)	1 (2.7)
Hypertension, n (%)	0 (0)
Pulmonary embolism, n (%)	1 (2.7)

Clinical characteristics presented as median (IQR) or absolute number (%).

*BSA* body surface area, *EVF* erythrocyte volume fraction, *Hs-TnT* high-sensitive troponin T, *PAP *pulmonary artery pressure.

^a^Data missing for n = 26.

**Table 2:** CMR findings of the patients.

CMR findings	n = 37
LVEDV, ml	158 [150–194]
LVEDVI, ml/m^2a^	79 [72–89]
LVESV, ml	73 [61–92]
LVESVI, ml/m^2a^	35 [29–43]
LVSV, ml	91 [73–102]
LVSVI, ml/m^2a^	45 [36–49]
LVEF, %	55 [49–59]
LVM, g	98 [74–120]
LVMI, g/m^2a^	46 [41–55]
Heart rate rest, bpm	70 [64–80]
Heart rate stress, bpm	89 [82–102]
ECV, %	25 [23–27]
LGE, n (%)	4 (11)

CMR findings are presented as median [IQR].

*CMR* cardiac magnetic resonance imaging, *LGE* late gadolinium enhancement, *LVEDV* left ventricular end-diastolic volume, *LVESV* left ventricular end-systolic volume, *LVSV* left ventricular stroke volume, *LVM* left ventricular mass, *LVEF* left ventricular ejection fraction, *bpm* beats per minute, *ECV* extracellular volume.

^a^LVEDV, LVESV, LVSV and LVM were indexed to BSA, which was calculated according to the Mosteller formula^17^.

**Table 3:** Rest and stress findings of the patients.

CMR findings	n = 37	Normal values^10^
Native T1, ms	1006 [983–1027]	998 (930–1050)
Native T2, ms^a^	48 [46–50]	48 (44–53)
Stress native T1, ms	1049 [1012–1073]	1049 (960–1140)
Stress native T2, ms^a^	52 [50–54]	56 (50–60)
∆T1, %	4.6 [3.0–6.3]	5.9 (1.3–11.0)
∆T2, %^†^	11.0 [3.8–14.0]	15 (4.4–26.0)
Myocardial rest perfusion, ml/min/g	0.89 [0.76–1.1]	0.9 (0.50–1.25)
Myocardial stress perfusion, ml/min/g	2.71 [2.27–3.39]	3.4 (2.19–5.04)
MPR	3.1 [2.7–3.6]	4.2 (2.2–6.3)

CMR findings are presented as median [IQR]. Reference range was calculated as mean ± 2 standard deviations from^10^.

*MPR* myocardial perfusion reserve.

^a^Data missing for n = 2.

### Relationship between native T1, native T2 and myocardial perfusion

**Figure 3:**
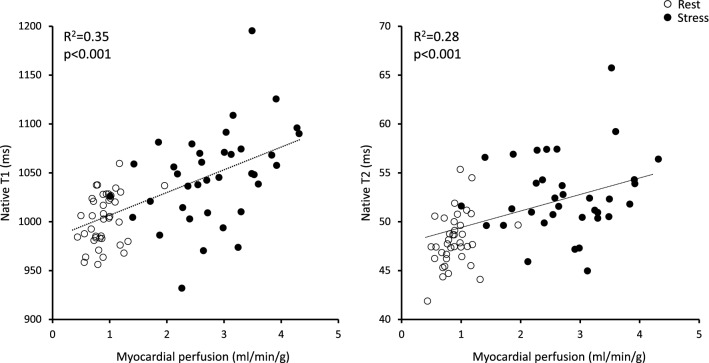
Relationship between rest and stress native T1 and T2 and absolute myocardial perfusion. Scatterplots comparing native T1 and T2 with absolute rest (white) and stress (black) myocardial perfusion.

In the analysis of pooled rest and stress data, myocardial perfusion correlated with both native T1 (R^2^ = 0.35, p < 0.001) and native T2 (R^2^ = 0.28, p < 0.001), respectively, Fig. 3. The relationships between native T1, T2 and myocardial perfusion at rest and stress, respectively, are presented in Table 4. Native T1 at rest correlated moderately with myocardial stress perfusion (R^2^ = 0.20, p < 0.01), but did not correlate with myocardial rest perfusion. Native T1 at stress correlated moderately with myocardial stress perfusion (R^2^ = 0.17, p = 0.01), but not with myocardial rest perfusion. Native T2 displayed no correlation with myocardial rest nor stress perfusion.

**Table 4:** Relationship of native T1 and T2 with myocardial rest and stress perfusion.

	Myocardial perfusion rest R^2^, p-value	Myocardial perfusion stress R^2^, p-value
Native T1 rest	0.03, p = 0.09	0.20, p < 0.01
Native T1 stress	0.03, p = 0.29	0.17, p = 0.01
Native T2 rest	0.05, p = 0.22	0.11, p = 0.06
Native T2 stress	0.005, p = 0.70	0.02, p = 0.40
∆T1	–	0.04, p = 0.26
∆T2	–	0.004, p = 0.71

### Relationship between ∆T1, ∆T2 and myocardial perfusion reserve

Native T1 and T2 did not correlate with MPR at rest nor stress, Table 5. ∆T1 and ∆T2 did not correlate with MPR.

**Table 5:** Relationship of native T1 and T2 with myocardial perfusion reserve.

	Myocardial perfusion reserve R^2^, p-value
Native T1 rest	0.04, p = 0.25
Native T1 stress	0.06, p = 0.16
Native T2 rest	0.005, p = 0.70
Native T2 stress	0.002, p = 0.79
∆T1	0.02, p = 0.36
∆T2	0.000, p = 0.99

### Reproducibility

Intra- and inter-observer agreement are presented in Table 6 as ICC. Overall, there was an excellent agreement for native T1, native T2 , myocardial perfusion, MPR, ΔT1 and ΔT2.

**Table 6:** Intra- and inter-observer variability presented as ICC and p-values.

	Native T1 rest	Native T2 rest	Myocardial perfusion rest	MPR	ΔT1	ΔT2
Intra-observer	0.92, p = 0.001	0.98, p < 0.001	0.99, p < 0.001	0.99, p < 0.001	0.96, p < 0.001	0.97, p < 0.001
Inter-observer	0.91, p<0.001	0.96, p < 0.001	0.95, p < 0.001	0.99, p < 0.001	0.95, p < 0.001	0.82, p < 0.001

*MPR* myocardial perfusion reserve.

## Discussion

We have demonstrated that both native T1 and native T2 correlate with myocardial perfusion, reflecting the coronary circulation during follow-up of patients with severe Covid-19. The recent trend towards the use of non-contrast techiques to detect changes in the coronary circulation needs to be elucidated in several different clinical contexts and with different clinical parameters, and in this study neither ΔT1 nor ΔT2 correlated to MPR. Therefore, native T1 and T2 mapping seem to capture changes in perfusion, however, the findings of ΔT1 and ΔT2 suggest that non-contrast methods may not be clinically applicable for diagnoses made with MPR, such as obstructive CAD or CMD.

### Relationship between myocardial perfusion, native T1 and native T2

The use of native T1 during adenosine stress to capture the intravascular compartment of myocardial perfusion was first illustrated by Mahmod et al. in a mechanistic study of patients with AS with a blunted ΔT1 that normalized following intervention^18^. ΔT1 as a possible diagnostic measure has been reproduced in both ischemic heart disease^19^ and diabetes^20^. However, due to the lack of a quantitative reference method for comparison, these mechanistic studies did not show the relationship between native T1 and myocardial perfusion. We elucidated the relationships between myocardial perfusion and native T1, and native T2, respectively, in normal physiology^10^, displaying the physiological basis for native T1 and native T2 for non-contrast diagnosis of the myocardial microcirculation, however more data in clinical contexts are needed. Previous experimental work in animals predicts that tissue T1 depends on perfusion and regional blood volume^21^ which is supported by work in humans^10,18,20^, however given the relative low perfusion in humans there are still big knowledge gaps for the translation of stress native T1 into the clinical work. Everaars et al. compared native T1 mapping with 15O(H2O)PET myocardial perfusion in patients with suspected CAD, and showed a moderate correlation between rest and stress measurements of native T1 and myocardial perfusion^19^. In normal physiology this correlation is stronger^10^, which is supported by the findings of the current study. A strong relationship between native T1 and myocardial perfusion was also found in healthy subjects undergoing regadenosone stress^22^. Myocardial stress perfusion is the net result of perfusion and increase of myocardial blood volume across the entire coronary vasculature which may explain the relationship between native stress T1 and myocardial stress perfusion in all three studies. However, there was no correlation between myocardial rest perfusion and native T1 at rest in patients with suspected CAD^19^, reproduced by the results of the current study, in contrast to normal physiology^10^. Potential contributing factors to the difference between patients and healthy volunteers besides age and distributions of sex^23^, include choice of native T1 mapping sequence, physiology in the presence of pathology, sample size and differences in contrast agents^24,25^.

Native T2 also correlated with myocardial perfusion, as previously shown in normal physiology^10^. Unlike native T1, individual T2 rest and T2 stress did not have a significant correlation with myocardial rest or stress perfusion in neither the present study nor in normal physiology^10^. More studies on native T2 during stress is needed to further understand the mechanisms behind non-contrast imaging for diagnostic use.

### Relationship between MPR, native T1 and native T2

Native T1 during stress has previously been shown to moderately correlate with MPR in diabetic patients^20^. However, this has not been reproduced in the current study, or in patients with CAD^19^, and AS^18^. The present study found no correlation between MPR and ΔT1, as previously reported^18,20^, while Everaars et al. found a weak correlation^19^. Furthermore, there was no correlation between ΔT2 and MPR in the current study, suggesting that non-contrast techniques cannot capture the MPR. It could be hypothesized that ΔT1 or ΔT2 do not reflect the same physiology as MPR. Native T1 and T2 increase with free water content, which may have an intracellular or extracellular origin, including the intravascular and interstitial compartments^18^. It is still unclear if the change in native T1 and T2 during stress is primarily dependent upon the changes in myocardial blood volume, ECV or myocardial perfusion. While myocardial perfusion is closely linked to myocardial blood volume and ECV, their intrinsic relationships have not been completely elucidated. Furthermore, native mapping may not be able to differentiate between myocardial blood volume and myocardial perfusion, and there may be a need for a contrast agent to separate the contributions of myocardial perfusion and the myocardial blood volume and ECV^10^. However, the correlation between pooled rest and stress values of myocardial perfusion and native T1, and native T2, respectively indicate that the underlying physiology behind the parameters are closely related.

### Comparison with controls

In Table 3 it is evident that this overall post-covid population are within the normal values, adding additional knowledge of cardiac pathology in long-term follow-up of severe covid-19. However, the scope of the current study was not to compare the CMR findings with controls, but rather display the heterogeneity in multiparametric native stress imaging in different populations to add the current body of knowledge of native stress mapping. Furthermore, looking at the normal values (mean ± standard deviations) of ∆T1 and ∆T2, the reference ranges with MOLLI 5 s(3 s)3 s are wide, thus limiting the diagnostic potential of stress native multiparametric mapping. In a recent PET study using regadenosone it was shown that patients with post acute sequele of covid-19 syndrome (PACS) had an impaired endothelial-dependent vasodilator response compared to controls^26^. It has previously been shown that ∆T1 are mediated through both endothelial and non-endothelial dependent mechanism, while adenosine perfusion and MPR are solely mediated through non-endothelial dependent mechanism^27^. Given that PACS patients more often are affected by postural ortostatic tachycardia syndrome (POTS) together with the observed differences in adenosine and regadenosone response with regards to vasodilator response this might explain the differences in results between the two studies. Further research into the response in ∆T1 and ∆T2 between adenosine and regadensone with regards to endothelial and non-endothelial dependent mechanisms are needed both in covid-19 but also other diseases.

### Clinical outlook

The correlation between pooled native T1, T2 and myocardial perfusion, shows that native T1 and T2 mapping indeed can be used to capture changes in myocardial perfusion during stress without the need of contrast agent. Standardization of native T1 mapping protocols remains important to mitigate cofounders such as age and sex. This study is to the best of our knowledge the first to investigate the relation between native T2 and myocardial perfusion in the presence of pathology and present the correlation between ΔT2 and MPR. While there is potential in non-contrast methods during stress for assessing the microcirculation, given that there is no correlation with MPR the diagnostic potential may be limited. Further research is needed in different patient groups, and with different native T1 mapping protocols.

### Limitations

In this prospective cohort study of previously healthy 37 patients from a single center was included, which is likely reflected in the structurally normal hearts of the study population. Only one native T1 and native T2 map, respectively, were acquired during stress, however there was no differences in significant correlations between one vs five slices at rest (data not shown). Furthermore, one slice at stress does not cover the entire ventricle, why it would be interesting to perform full coverage native mapping in the future. Another limitation is the heart rate dependency associated with native mapping techniques, where Shortened MOLLI and MOLLI 5 s(3 s)3 s (used in this this study) are two different techniques to mitigate heart rate dependecy compared to other MOLLI sequences^28^.

## Conclusions

Native T1 and native T2 correlate with myocardial perfusion during adenosine stress, reflecting the coronary circulation in patients during long-term follow-up of severe Covid-19. Neither ΔT1 nor ΔT2 can be used to assess MPR in patients with severe Covid-19.

## Data Availability

The data that supports the findings of this study is available from corresponding author upon reasonable request.

## Abbreviations

AS: Aortic stenosis
CMD: Coronary microvascular dysfunction
CMR: Cardiac magnetic resonance imaging
Covid-19: Coronavirus disease 2019
CVD: Cardiovascular disease
ECV: Extracellular volume
FA: Flip angle
FOV: Field of view
Gd: Gadobutrol
hs-TnT: High sensitive Troponin T
LGE: Late gadolinium enhancement
LVEDV: Left ventricular end-diastolic volume
LVEF: Left ventricular ejection fraction
LVM: Left ventricular mass
LVSV: Left ventricular stroke volume
MPR: Myocardial perfusion reserve
MOLLI: Modified Look-Locker Inversion recovery
PAP: Pulmonary artery pressure
PACS: Post acute sequele of covid-19 syndrome
PET: Positron emission tomography
POTS: Postural ortostatic tachycardia syndrome
SSFP: Single shot steady state free precession
TD: Trigger delay
TE: Echo time
TR: Repetition time
